# Essay upon Professional Fees

**Published:** 1856-12

**Authors:** Elisha Townsend


### ESSAY UPON PROFESSIONAL FEES,

BY ELISHA TOWNSEND, D. D. S., M. D.

READ BEFORE THE AMERICAN DENTAL CONVENTION AT THEIR SECOND ANNUAL MEETING IN AUGUST, 1856.

Four years ago I had the honor, by appointment of the American Society of Dental Surgeons, to read before it a paper upon Professional Fees.

Constitutionally averse to those forms and bearings of controversy which have anything of moral assault and battery in them, I forebore to press the just complaint of the Profession against fees exceptionably low, for, though I could be sure enough for my own satisfaction, that the deserved animadversion would be free from all personal feeling on my own part, and just as free from any intended personal application to the individuals justly falling under the censure, I could not be sure that it would escape such application, and the unpleasantness of feeling that follows hard-hitting, however free from animosity. Feeling since, that the truths which govern conduct in this matter, ought to be driven clean through the subject and clinched on the other side, I propose now to add a few strokes of the hammer, let the point turn and grip where it may.

*I* give it no special direction, and if it pinches, or goes against the grain, or makes a split, I will only have to pay for the putty that will smooth it over again.

I could be well contented to leave the wrong to exhaust itself, while only inferior men, knowing no better, and capable of nothing better, work at handicraft-wages for such work as they sufficiently reward; because, I suppose, the wood-sawyer does not seriously injure or dishonor the cabinet maker. The tools which they respectively use and the sawdust which they make, sufficiently distinguish them for the security of the superior artizan's interest and reputation, and there is no danger of the shop being confounded in public apprehension with the curbstone; but, when the mechanic, free of his guild, compromises with the cross-cut and the wood-horse, and *cords* up his jobs with a roughness proportioned to his speed, and lowers his prices a little and increases his business a great deal, to the injury and discredit of his art—then I think his brother chips have a matter of just complaint against him. The grounds of this complaint are these, and the like: Workmen with a good deal of skill, can botch jobs, in all points capable of concealment from their customers, to an extent that greatly diminishes the cost of production, and as greatly under-bids first-rate articles of the same kind in the market; and the reputation of the jobber, if he holds a good professional position, adds a false warranty to the quality, and so drives fair and honorable competition out of the field.

A man's real superiority in his art is his lawful advantage; he ought to have it; and the world should have the benefit of it, and his competitors are benefitted by all their efforts to work up to him. Nothing but good grows out of such excellence to any body whom it affects. It is all fair trade, but, the tricks of trade are another matter, and stand to a different account, which his fellow craftsmen are concerned to settle against the man who employs them on the ground that their just rights are violated by such unfairness.

Sleight of hand, in its honest English sense, is lawful, but Frenched into legerdemain it is contraband, by all the rules of public policy and private rights. The inspection laws of the State and the city are grounded on the principle of the public protection and the defence of the fair dealer against fraudulent competition, and the moral sentiment which condemns false measures and false weights, justly bears still heavier against false appearances which are more dangerous and mischievous by all the difference of difficulty in their detection. Besides the injury to private rights, there is a great wrong inflicted upon the craft by all successes of unfair dexterity, which all parties are deeply concerned to prevent.

If excellence in an art, and progress in its improvement are rendered unprofitable, and their conditions impossible by workmanship that tends to deterioration in the quality of the product, and the undiscerning and incapable public are seduced into its encouragement, the craft is demoralized, and all genius and skill that are honest are driven out of it, as all honorable eminence is prevented in it.

This is bad even for the ancient and honorable company of wood-sawyers, so far as tricks are possible in their trade; but, when it creeps up into the range of art where veneering and imitation oaks, walnut, and cherries are practicable, all dexterity at deception and underselling in the market puts the business more and more within the province of the craftsmen's concern.

Out of the relative rights and duties which the industrial arts create among their members, the necessity for friendly, fraternal, and business associations arises.

In Continental Europe, *Devoirs* for all the trades, associate the workmen into companies, whose constitutions rule the whole business conduct of the acknowledged membership; and, under the rudely administered justice of the fraternities, the men who offend against the common interest and honor, get their heads broke by way of caution to the weak brethern who are in danger of being subdued or seduced by the misconduct of the unworthy.

Free-masonry probably had its origin in this policy of a handicraft, when its hammer, plummet, and trowel were the implements in actual service. Our own printers, carpenters, and other trade-unions, are of the same character, and have similar objects though differently effected in the infliction of penalties.

And it is worthy of notice that while all the conduct of the craftsmen which affects the brotherhood is put under the government of their unions, as it ought to be, the main and ruling idea of them all is, the regulation of prices, as the means of securing every other beneficial object in their aim. There is a sound philosophy in this idea, which I need not discuss farther than just to observe that under the rule of low wages, proficiency is levelled to the rank of unskilfulness, eminence is starved down to mediocrity, or driven out of employment, and the men who have in good faith learned an art, find that learning useless to them, and they must either go back again in all their conditions to the society of the unlearned, or use their skill to abuse the public confidence, and destroy the best interests of an honorable calling.

Under-working and under-selling having these consequences may well be regarded as treason to trade, and mortal sins in the decalogue of business morals.

The law of master-dom long treated such associations as conspiracies and punished them as misdemeanors, with fine and imprisonment, but revolutions in the civil and social systems of society, have settled the justice of such associations, purely upon their necessity; and the legitimate sovereignty of the useful classes has dethroned the despotism of the aristocracies of wealth and civil power.

The workers who make the world's wealth have grown strong enough upon their small share of it, to obtain the government of the bread question, and knowing that all other rights and liberties must begin there, they say that whoever touches their prices to depress them, is their enemy ; and if one of themselves is the offender he is a traitor, and must be treated as one, with no other respect of persons than an increase in the penalty in proportion to the mischief he is capable of doing. These associates in hard work and partners in a common interest have other ways of lynching a delinquent than by violence—they wont work with him, nor consent by any act of tolerance to help the mischief that he does them. They are right in the principle, and are only in danger of mistake in its application. This danger of theirs is owing chiefly to the fact that the administration of discipline is necessarily in the hands of the injured, outraged and impassioned party, and so may catch the character of selfish revenge, and carry the punishment to undue and unnecessary lengths.

This is not so in our profession—it is in the power of the men who are not at all affected in their private interests to apply the remedy. No man has so little personal concern in quackery as the well established and well educated physician ; *his* zeal for the repression of the mischief grows out of his reverence for his profession and his solicitude for its advancement in usefulness and honor, and he can therefore be trusted to exert all his legitimate force in the correction of the mischief. So well, and so faithfully, do the members of the professions long and well-established, exert this power in their hands, that all the men, in regular standing with them, are held up to the common standard of conduct, both in the etiquette of practice and the rate of charges on which the common character and general prosperity of the faculty depend. The want of such conformity prevailing in any learned and liberal art to any considerable degree, is a sign of its immaturity and disorderliness. It proves, not the absence of the sentiment among its members, but the lack of an effectively exerted opinion for the prevention of abuses.

The Dentists of this country have not yet been able to organise themselves sufficiently for that combination of action upon the practice which would compel a general observance of duty by all the practioners in tolerable standing with the community; but it is the time now, in my judgment, to take ground, and endeavor a reformation with the hope of a very early success.

The men who meet here in convention are sufficient to begin the work, so that it must be soon and satisfactorily accomplished. Let us make it the subject of reflection and discussion, and so set the forces at our command in motion. We have already with a success both earlier and greater than any of us would have ventured to promise five years ago, liberalized the societary influence of the fraternity, and now it is doubly imperative upon us to see that the improvement of our profession's conditions shall keep pace with its enlarged freedom of intercourse and association.

We have made a successful movement for the *freedom* of the fraternity, and we must next strike for its honor and well-being. This is fairly within the power of a well-digested opinion upon professional duty and a corresponding enforcement by all the means and influences which an established sentiment can command.

We are not calling for tests, pledges, or quack laws of our own contrivance, but for the authoritative expression of the better judgement of the fraternity concerning the duties of the practitioner to the profession, and the resulting determination to carry it into effect by all honorable means within the power of each individual convinced of its propriety and obligation.

I think, and others whose names would greatly strengthen the opinion, think with me, that now is the time for action on the subject. Some of the reasons which support the opinion may be given, but without pretending to exhaust them or to cover the whole ground of the exigency.

The system of education in Dentistry which may be called classical, is rapidly rising in estimation; elementary treatises, periodical literature, and collegiate instruction are multiplying their force and efficiency at a rapid rate. The standard of requirement is rising in and out of the profession in due proportion to these powerful agencies, and a laudable ambition for excellence is growing to be the characteristic of the pupils and younger aspirants for professional success. To gratify and reward such aspirations where they exist, and to invite that sort of spirit, by providing for its indulgence and rewards is therefore a special duty of the time.

It is indeed the duty of all times, but it is the necessity of the present; and we must address ourselves to it.

Through all the public agencies of the educational function, there is but one tone and one tendency—the improvement of our art: the stimulus and the exhortation are as impulsive and impressive as they can be made, but what can promptings and precepts avail when they are resisted by agencies which have the power to compel the surrender of principle, or crush the prosperity of the generous young man who endeavors to maintain it?

If the men who have an established reputation degrade prices below such remuneration for good work as the young candidate for business success can live by, the struggle to which he is put may be something more than he can sustain—when he is told by every applicant that comes to him with a huckstering spirit, that Dr. somebody, that everybody knows and has more patients than he can attend to, charges but little more than half the prices which he asks; what shall he say to it? and what shall he do about it? He has the skill and science of the profession, but he has yet no fame which can command the business of the more judicious and liberal class of patients, and he must succumb or submit: he must substitute speed for perfection of work, he must contrive to just satisfy a patient who has learned that the thing can be done for a price which he has every reason to believe is compensation for standard services, and, if he consents, he has done that which will make every accommodation afterwards easy in proportion to its practicability.

He must do up twice as many cases, put in twice as many fillings as he can do well, or he can and must do nothing; and if he is driven to under-working, and under-bidding the man who first put him upon the necessity, it is well, or rather it is not quite so bad as it might be.

Now the men who are engaged in this practice deserve to be told that they are engaged in degrading the profession, and ought, by the just rules of Medical and Dental etiquette, to be ruled out of it. Whoever is concerned to keep up its honor and reputation, is just as much concerned to put them down, and will, in all their professional conduct accordingly give them a *tremendous letting alone.*

They will say to him, we have seen other quackeries subside upon experience and with one voice we intend to settle yours in the same way. We made them open and declared enemies of the fraternity and just as soon as we can get you to put on so much honesty, your case is settled. We do not impeach your ability, but your professional honor, and one of these days your intelligent patients will be down on you like judgment day, and you may then fall back on the lane and alley practice which you have so assiduously courted. The older men care nothing at all about you now, and very soon you will be out of the way of every honorable boy in the brotherhood.

These cheapeners of talents and skill have, beside their direct depressing power upon beginners, a pernicious influence over them. They are usually great boasters about not only the number of patients which they have, but they talk like the fast young man who said, "he ran through Shakespiere one day and Euclid the next, skipping what he called the 'figgers,' in the the one, and the long speeches in the other." Speed,—speed,—is their cry, as if it were acres of ground they were ploughing through, instead of achers of dentine they are concerned with—and they have as many reasons to give, (reserving always the true ones) for rushing through or over their work, as may satisfy any fool, and some novices in the practice. They do not seem to recollect that whoever works well enough is certain to work fast enough, and also that the character of the service is to be settled by the duration of the benefit, and not by the speed of its execution.

The man that can utter fifty words more in a minute than anybody else, might as well offer the fact to prove that he is a capital elocutionist, and the unhappy subjects of the gabble would be about in the same predicament with those that are the patients under such a scuffle of the dentist with time for the stake of a dollar.

I know not what amount of celerity in the finger-smithing of our art may be attained by those who aim at nothing else, and I know no use that such curious information would serve; I would look for, and could appreciate, such qualities in a sewing machine, or a nail-cutter, or a power-loom; but what has it to do with filling a tooth, or with fulfilling the Dentist's real duty to his patients? "Slow, but sure," would probably never have got itself into a proverb, but for the impudent pretentiousness of that heels-over-head-haste which it serves to rebuke.

Slowness and fastness alike are entitled to no consideration on their own account. Skill and integrity are not concerned about either; with them the question is, quality of execution, and time must take care of itself in the process, for it is of no account when the substance of the duty is secured.

The fast man is too fast to be trusted, the slow man too slow to be endured, the capable and honest man is neither the one nor the other, nor is he in any way concerned with the question of velocity, except as it is involved in the perfection of his work.

The subject of professional remuneration, it is admitted, is a difficult one to get into a fee bill. Rules would make it too stiff, and arithmetic too inflexible, but principles can govern it safely. Patients should pay for the skill that they need and the advancement of the profession which has charge of their interests, and that pay should be a fair bid for the best talents and the most devoted attention which is required ; and whoever offers less, and whoever offers to accept less, deserve to know and to feel that they are unworthy of the services and of the ministries of the art.

Extortion of exorbitant prices is a sin against the trust reposed in a professional man by his patient, and in the last degree dishonorable to the one and injurious to the other, and no man but a ridiculous egotist or detestable miser, would so pick a pocket that is frankly opened to him. But this much may be said even for extra high charges in Dentistry, that every body may know the rate, and must be about as well persuaded as the operator that it is a good enough bargain before it will be made ; while on the other hand, the extra low charge is made the main inducement, and the operator is the only party to that bargain who certainly knows it to be a bad one for the patient, and so the money motive falls along with the prices, heavier the lower they descend. Lynching is the revenge of a rude system for the offence, and contempt is its correspondent in a higher range of life for the same thing—the offence being so much the greater, justifies the greater severity of the penalty—for, I take it an honorable man's scorn is heavier and severer than a short ride on a rail.

Here I very willingly leave the less agreeable subject of these strictures and turn to a point in professional conduct that, perhaps, as much as any other demands the attention of the faculty. I allude to counsel fees—charges for advice to our patients when no other service is rendered to them. In medical practice advice is *the* thing charged for. Whenever the physician is not his own apothecary, he has nothing else in his apprehension in making out his bill. If the account reads so many visits, so many dollars, though the word "*consilium*," happens not to occur among the items, you may depend upon it the doctor does not intend to be understood as charging only for the services of a runner, or messenger, or servant to his patient—one, or two, or three visits means just one or two or three advices, as many instances of skill and judgment in the case put at the service of the patient ; and however time and distance may figure in the account, it is not pedestrianism or wages by the day or hour that is the substance of the charge—he never thinks of his muscular service as anything but a necessary incident to the exercise of the professional function, and names it only as a means of measuring the value of the science and skill exerted in behalf of his patient.

Moreover, he always charges *advice*, and that in proportion to its intrinsic value, charges it nakedly and independently when neither time nor toil make a sufficient figure in the service to cover its fair remuneration.

This is the difference between a learned profession and a mere handicraft. And, if Dentists intend to give their branch of the remedial art a fair position among the liberal arts, they must openly and distinctively estimate what learning and skill they have, and demand for the service of their brains a fair remuneration, and also secure for them a direct acknowledgement by their patients and by the public. A gentleman who has some time or other consulted a physician, surgeon, or lawyer, and knows what that intangible thing called professional advice is worth to him, and at what expense of time and toil and talent the power to give it has been acquired, takes his seat in a dentist's chair, his teeth are carefully examined, and without any handicraft services, he is advised to his benefit and assurance, and the dentist, because he has done nothing with his fingers, is as shy of charging for his professional judgment as if either his art were not a profession, or he could not modestly claim to belong to it, and therefore the conclusion is a settled one, that his office is a shop, and his art a trade—by his own showing.

To those among us who have some consciousness that this is below the dignity of our profession, but are without the courage to maintain it, I would say as Hamlet says to the poor players, who having neither the accent or gait of physician, surgeon, or dentist, but play a sort of journeyman character in the business: "Oh, reform it altogether."

I say charge for your advice, charge for the thing that your patients require of you, on the grounds of their confidence in you, and deliver your calling from a degrading depreciation. You know the claim to be well founded—make your patients know it as well. It is the very thing they need to know, and their faith in it is the most valuable thing to them of all that their money can possibly purchase from you.

Some of us here present have lived through all the painful period of our profession's growth from the lowest rank in public estimation till it has secured a reputation level with its own highest demands.

It is the youngest branch of a respectable family ; in its childhood it was bound out to service, but its blood is gentle, its instincts noble, and it has only now in its well grown manhood to assert its natural rank and so secure it. Already it stands as a profession in the average clearer of quackery and incompetency than law, medicine or theology. Let its practitioners but walk worthy the calling wherewith they are called, and its public honors will very speedily answer like an echo to its just pretensions.

I need not press upon such an audience as this, the argument which concerns our own advancement, through the reactive influence of the fraternity. Every man capable of weighing the force of a professional standard, the influence of a professional ideal, acting upon the mass of its practitioners, knows better than he or I can explain, how mighty the power of opinion is, in the reformation of abuses, when that opinion touches the spirit-springs of enterprise and progress ; it is the trumpet charge that wakens the dullest dragoon to duty, and makes a hero of every common soldier. He looks to the floating standard of the corps, and the corporate enthusiasm carries him clearly abreast with the most generous spirit in the ranks.

I am speaking now to the representatives of our rising profession, and I know that neither hope nor faith is wasted on them. Upward, onward, is the impulse that is stirring in every heart, and there is not a laggard in all the ranks that *can* check our progress now. "We count not as though we had already attained, neither were always perfect, but this one thing we do —forgetting those things which are behind, and reaching forth unto those things which are before, we press toward the mark for the prize of our high calling in liberal learning."

